# Gnathic Bones and Hyperparathyroidism: A Review on the Metabolic Bony Changes Affecting the Mandible and Maxilla in case of Hyperparathyroidism

**DOI:** 10.1155/2020/6836123

**Published:** 2020-07-09

**Authors:** Hazim Mahmoud Ibrahem

**Affiliations:** Department of Basic Sciences, Al-Mustansiriyah University, College of Dentistry, Baghdad, Iraq

## Abstract

Parathyroid glands secrete the parathyroid hormone that plays an essential role in bone remodeling. Excessive production of parathyroid hormone causes a common metabolic bone disorder known as hyperparathyroidism that is classified into primary, secondary, or tertiary. In hyperparathyroidism, the late bony complication is manifested as a giant cell osteolytic lesion called “brown tumor.” Primary hyperparathyroidism is usually a sporadic disorder, but in minority of cases it occurs in inherited forms, and one of these forms is the hyperparathyroidism-jaw tumor syndrome, which is characterized by primary hyperparathyroidism and ossifying fibroma in the mandible and/or maxilla.

## 1. Introduction

There are 4 parathyroid glands located behind the thyroid gland which secrete parathyroid hormone “parathormone” (PTH) which maintains a proper calcium balance in bloodstream and in tissues that require calcium for their physiological functions. Excessive secretion of PTH and prolonged exposure of bone to PTH result in the metabolic bone disorder known as hyperparathyroidism (HPT) [1]. Brown tumor is comparatively an uncommon non-neoplastic osteolytic lesion of bones that appears in advanced stage of hyperparathyroidism [2]. Association of primary hyperparathyroidism and ossifying fibroma of the jaw is seen in a rare hereditary syndrome referred as hyperparathyroidism-jaw tumor (HPT-JT) [3].

This review focuses on the gnathic metabolic bone changes that result from increased parathyroid hormone secretion in case of hyperparathyroidism.

## 2. Review

### 2.1. Hyperparathyroidism

Hyperparathyroidism could be described as an endocrine disorder resulting from increased secretion of parathyroid hormone and is characterized by hypercalcemia due to increased mobilization of calcium from bone to circulation [4]. HPT is classified into the following clinical forms:

1. Primary HPT (PHPT) which is either of a nonfamilial (sporadic) form representing about 90–95% of all PHPT or familial inherited form representing 5–10% of PHPT [5].
2. Secondary HPT which results from a primary condition producing hypocalcemia such as rickets, osteomalacia, vitamin D deficiency, prolonged hypocalcemia caused by chronic renal failure, and long-term dialysis [6, 7].
3. Tertiary HPT which results from long-standing secondary HPT [8].
4. A fourth type of HPT (because of ectopic parathyroid gland) which is thought to arise in patients with malignant diseases [9].

Clinically, HPT shows variation in signs and symptoms including bone fractures, kidney stones, peptic ulcers, easy fatigue, sense of weakness, depression, anxiety, and myocardial, valvular, and vascular calcification [10, 11].

### 2.2. Metabolic Bone Changes in Hyperparathyroidism

In HPT, bone metabolic changes are considered as the late manifestation, and these include bone erosion, bone resorption, and localized bone cystic lesions named as “brown tumors.” Together, all these features are described as osteitis fibrosa cystica [12, 13].

#### 2.2.1. Brown Tumor

Brown tumor (BT) is non-neoplastic giant cell lesion that appears in advanced stage of hyperparathyroidism. It represents a giant cell reparative cellular phenomenon that involves areas with intense bone resorption due to the effect of high circulating PTH levels [14].

The real global incidence of brown tumor is not well estimated [15]. With the development of imaging and laboratory screening methods, hypercalcemia in cases of HPT can often be discovered in early stages resulting in a decline in the frequency of brown tumors [16]. All the information related to the prevalence and incidence of brown tumors are dated at the end of 1990s and have not been updated, and these information indicated that brown tumor frequency is of 3–4% in primary hyperparathyroidism and 1.5–1.75% in secondary hyperparathyroidism [17]. In maxillofacial region, more than 70% of the lesions affect the mandible while very few cases have been reported in the maxilla [18].

The incidence of brown tumor is more in people older than 50, and the female: male ratio is 3 : 1 [19].

The term “tumor” is a misnomer because the lesion does not have a neoplastic potential, and any bone may be involved by this lesion, though the common locations are the long bones, ribs, clavicle, spine, extremities, pelvic girdle, mandible, and maxilla with the mandible being more affected than maxilla [20, 21].

Pathological increased level of PTH in HPT causes increased resorption of bone trabeculae in a “tunneling” or “dissecting” pattern which leads to the enlargement of marrow space causing weakening of the bone, and this weakening will lead to microfractures and intraosseous microhemorrhages. Microfractures, intraosseous bleeding, and tissue degeneration cause influx of macrophages ending with hemosiderin-loaded macrophages, giant cells, and fibroblast filling the newly formed small osteolytic cysts. These small cysts fuse together giving the characteristic brown color of this lesion [22, 23].

Clinical symptoms caused by brown tumors depend on their location and size; they range from small asymptomatic lesion, discovered accidentally by radiological examination, to a large locally destructive lesion resulting in a variety of symptoms that are mostly related to facial deformation and disfiguration, such as difficulty in chewing, talking, and breathing. Intraorally, it presents as an expansile bone lesion (localized or diffuse); it causes abnormal occlusion due to mobility and displacement of teeth in the affected region [24, 25].

Histopathological examination reveals a benign process. Brown tumor is considered as a kind of giant cell lesion that presents as an osteolytic lesion of the bone. It is nonencapsulated and characterized by multiple giant cells mixed with vascular fibrous tissue stroma, spindle-shaped mononuclear cells, haemosiderin-laden macrophages, and matrix containing large amount of haemosiderin [26].

The radiological appearance of the lesions may vary according to the stage of HPT. In some cases, they are poorly defined, while others have a sclerotic margin [27]. In hyperparathyroidism, the radiographic image of the skull shows “salt-and-pepper” appearance due to the trabecular bone resorption [28]. Brown tumors appear as well demarcated unilocular or multilocular radiolucencies with the bone trabecular pattern showing a “ground-glass” appearance. Oral radiographic manifestations associated with BTs include root resorption with generalized loss of lamina dura surrounding the roots of the teeth, loss of cortication around the inferior alveolar canal and maxillary sinus, and alteration of the trabecular pattern of the jaws [29, 30]. On CT scan, brown tumors mostly appear as a lytic expansive lesion, without periosteal reaction [31]. On magnetic resonance imaging, the image demonstrates hypo- or isointense on T1 and T2-weighted imaging, as well as a strong homogenous enhancement with gadolinium [32]. Thus, when a patient, especially between the age of 20–40 years, presents with unexplained osteolytic radiolucent bone lesions, radiologists should consider PHPT in differential diagnosis.

In differential diagnosis of brown tumor with a solitary bone lesion, it may be difficult to differentiate from aneurysmal bone cyst, true giant cell tumor, solitary bone cyst, cherubism, or giant cell reparative granuloma. In cases of multiple brown tumors, the differential diagnosis includes multiple nonossifying fibroma, osteolytic metastasis, multiple myeloma, multiple bone cysts, Langerhans' cell histiocytosis, metabolic osteopathy, and fibrous dysplasia [33].

Brown tumor fine needle aspiration smears show scattered giant and mononuclear cells. The mononuclear cells have scanty ill-defined cytoplasm with irregular margins and monomorphic nuclei. The giant cells are very scanty in number and may vary in size [34].

Management of brown tumor is often directed toward the normalization of PTH level through the management of the underlying cause of HPT, and this frequently results in regression and resolution of these lesions without the need of extensive surgical intervention; however, surgical treatment may be required in refractory cases or in large disfiguring symptomatic lesions that cause bone weakening [35, 36]. Surgical excision of the jaw lesion, when required, is usually done after parathyroid surgery [37].

### 2.3. Hereditary Hyperparathyroidism Syndromes

Most cases of primary hyperparathyroidism are sporadic, but in less than 10%, they are familial inherited in nature which are usually present as an autosomal dominant trait, namely, multiple endocrine neoplasia type 1 (MEN1), MEN2A, MEN4, familial hypocalciuric hypercalcemia (FHH), neonatal severe hyperparathyroidism (NSHPT), autosomal dominant moderate hyperparathyroidism (ADMH), hyperparathyroidism-jaw tumor syndrome (HPT-JT), and familial isolated hyperparathyroidism (FIHPT) [38].

HPT-JT Syndrome is a rare autosomal dominantly inherited disorder characterized by primary HPT caused mostly by parathyroid adenomas and fibro-osseous tumors of the mandible and/or the maxilla named ossifying fibroma [39].

HPT-JT is a complex disease with clinical diversity and long time span; it is characterized by two main categories, hyperparathyroidism and ossifying fibromas of the mandible or maxilla. Moreover, this disease could be associated with Wilms tumor, renal hamartomas, and kidney lesions [40]. The onset of primary hyperparathyroidism in HPT-JT syndrome occurs in late adolescence or early adulthood with average age 32 years [41].

Pathogenetically, HPT-JT syndrome is usually caused by mutations in the tumor suppressor gene named CDC73 (Cell Division Cycle Protein 73) gene which is previously known as HRPT2 (Hereditary Parathyroid Type 2) gene [42]. Germline mutations in CDC73 have been identified in most (about 50–75%) of HPT-JT kindreds [43]. Physiologically, CDC73 gene is responsible for the synthesis of nuclear protein parafibromin which is responsible for the regulation of cell growth and proliferation having antiproliferative properties, enhancing cell cycle arrest [44]. Female individuals with this syndrome may have benign or malignant uterine tumors [45]. HPT-JT is clinically diagnosed in individuals with any of the following characteristics: (1) PHPT and ossifying fibroma of the jaw, (2) PHPT and a close relative with HPT-JT, or (3) ossifying fibroma of the jaw and a close relative with HPT-JT [46].

#### 2.3.1. Associate Jaw Lesion (Ossifying Fibroma) in Hyperparathyroidism-Jaw Tumor Syndrome

The jaw lesion associated with HPT-JT syndrome has been reported to be histologically distinct from BT and does not resolve after parathyroidectomy, and it falls within the category of fibro-osseous lesions and identifies as ossifying fibroma of the mandible and/or maxilla [47].

It occurs in 25–50% of individuals with HPT-JT, and these jaw tumors may occasionally precede the development of hyperparathyroidism in HPT-JT patients by several decades [48].

*(1) Ossifying Fibroma*. Ossifying fibroma is a rare benign fibro-osseous neoplasm affecting jaw bone and is characterized by replacement of normal bone by a combination of fibrous tissue and newly formed calcified tissues of bone and/or cementum-like material [49].

Ossifying fibroma occurrence begins between the ages of 10 and 20 years, and females are affected more often than males [50].

Ossifying fibromas mostly involve only one side of either the mandible or maxilla, and the mandible is more affected than the maxilla. Multifocal ossifying fibromas are much less common [51]. Regarding the histogenesis and pathogenesis of OF, the majority of authors affirm that OF is periodontal in origin in which there is excessive proliferation of the mesenchymal cells of periodontal ligament. On the other hand, two studies suggest that the histogenesis of OF consist of two possible origins: first is periodontal in origin in which there is excessive proliferation of mesenchymal cells of the periodontal ligament and second is nonperiodontal in origin resulting from metaplastic process occurring in the connective tissue cells, the former being more common [52, 53]. Error in the process of odontogenesis may cause extensive mesenchymal cellular induction of periodontal tissue with the formation of new fibrous tissue, bone, and cementum, and those will lead to the development of ossifying fibroma in the jaw bone [54]. Precise triggering factors are still not known, but some suggested some factors that include trauma, previous extractions, and pre-existence of periodontitis [55].

Pimenta et al.[56] suggested that some cases of OF may arise as a result of somatic mutation in HRPT2 due to haploinsufficiency of the gene. de Mesquita Netto et al. [57] concluded that the contribution of HRPT2 gene inactivation to the pathogenesis of OF is marginal at best and may be limited to the progression of the tumor rather than its initiation. Chen et al. [58] suggested that CDC73 mutations are rare in sporadic OFs of the jaws, yet CDC73 may play a role in the pathogenesis of a small subset of tumors.

Clinically, OF can present as a single solitary lesion or rarely as multiple lesions and may occasionally be bilateral/multifocal [59, 60]. These tumors are slowly growing lesions that can grow to a size that causes the expansion of the buccal and lingual plates; they can also cause early tooth displacement and disruption in normal dentition, but typically, they do not cause root resorption and the lamina dura of involved teeth is usually missing; furthermore, the lesion may grow to an extensive size pressing on the adjacent vital structures such as nerves causing pain and paresthesia [61–63].

Histologically, ossifying fibroma is described as a demarcated and occasionally encapsulated benign neoplasm characterized by the replacement of normal bone by fibrous tissue and varying amount of newly formed bone and/or cementum-like material [64].

Radiological finding suggests that the lesion is usually well circumscribed having a sclerotic rim and demarcated from surrounding bone and shows a centrifugal growth pattern rather than a linear one [65]. On plain radiographic film, the appearance of OF depends on the stage of maturation of the lesion and the amount of mineralized tissue within the tumor; lesion may appear completely radiolucent, radiolucent with variable degrees of radiopacity, or totally radiopaque [66]. On CT, ossifying fibroma usually reveals a sharply circumscribed eggshell-thin rim of bone surrounding a lytic area and on MR T1-weighted imaging; they return low to intermediate signal, the low signal areas reflecting the osseous component. On T2-weighted sequences, ossified areas appear with low signal, while fibrous tissue exhibits a hypointense signal. The thick outer layer of the tumor enhances following administration of contrast [67]. The differential diagnoses involving radiolucent lesions include odontogenic cysts, ameloblastoma, central giant cell lesions, chronic apical periodontitis, and idiopathic bone cavity. Radiopaque lesions include fibrous dysplasia complex odontoma, idiopathic osteosclerosis, and cementoblastoma. Mixed lesions include osteoblastoma, calcifying cystic odontogenic tumor, and calcifying epithelial odontogenic tumor [68].

The treatment plan of ossifying fibroma is individualized and case specific, depending on the size, location, and nature and growth behavior of the lesion. Generally, the management comprises complete surgical excision of the jaw lesion till the healthy bone margin is reached, and bone grafting and reconstruction is to be considered when necessary, and this should be done after removal of the parathyroid lesions (parathyroidectomy) [69, 70]. OF tends to recur with incomplete resection, and the recurrence is more common in younger patients; thus, orthopantomography of the face should be preferably performed once in every three years to detect any possible changes with follow-up period of at least 10 years [71, 72].

Prognosis of ossifying fibroma appears to be good. There is no evidence suggesting that OF can undergo malignant potential [73].

## 3. Conclusion

Hyperparathyroidism is a metabolic endocrine disorder characterized by increased bone turnover. The osteolytic jaw bone lesions associated with hyperparathyroidism make up diverse disorders of which some are neoplastic or non-neoplastic and hereditary or nonhereditary, and some affect the elderly while others affect the adolescent and young adult.
